# Supplementary material for: Gating and noelin clustering of native Ca2+-permeable AMPA receptors
Source: Nature. 2025 Jun 23;645(8080):526–34. doi: 10.1038/s41586-025-09289-0 (PMC12422955; doi:10.1038/s41586-025-09289-0)
Supplement: Supplementary file 1 — This file contains Supplementary Figs. 1–8 and Supplementary Table 1. [file 41586_2025_9289_MOESM1_ESM.pdf]

nature portfolio

https://doi.org/10.1038/s41586-025-09289-0

Supplementary information

Gating and noelin clustering of native Ca^{2+}-permeable AMPA receptors

In the format provided by the authors and unedited

Gating and noelin clustering of native Ca^{2+}-permeable AMPA receptors

Chengli Fang[1], Cathy J. Spangler[1], Jumi Park[1], Natalie Sheldon[1,3], Laurence O. Trussell[1,2], and Eric Gouaux[1,3,*]

[1] Vollum Institute, Oregon Health & Science University, Portland, OR 97239, USA.

[2] Oregon Hearing Research Center, Oregon Health & Science University, Portland, OR 97239, USA.

[3] Howard Hughes Medical Institute, Oregon Health & Science University, Portland, OR 97239, USA.

*Correspondence to: Eric Gouaux, gouauxe@ohsu.edu.

SUPPLEMENTARY INFORMATION

Supplementary Fig. 1. Characterization of monoclonal antibodies 4H9 and 7D8.

Supplementary Fig. 2. The sequence alignment of human GluA1-4 with rat GluA1-4.

Supplementary Fig. 3. The raw western blot images.

Supplementary Fig. 4. The structures of GluA2-containing AMPARs in complexes with auxiliary subunits.

Supplementary Fig. 5. Cryo-EM analysis of recombinant GluA4 with Noe 1.

Supplementary Fig. 6. The sequence and FSEC profile for the engineered dimer of Noe 1 protein (dNoe 1).

Supplementary Fig. 7. GluA1-4 alignments at Noe 1 interface.

Supplementary Fig. 8. The FSEC profile for recombinant GluA4 and cerebellar CP-AMPAR, relative to Fig. 6.

Supplementary Table 1. The constructs in this study.

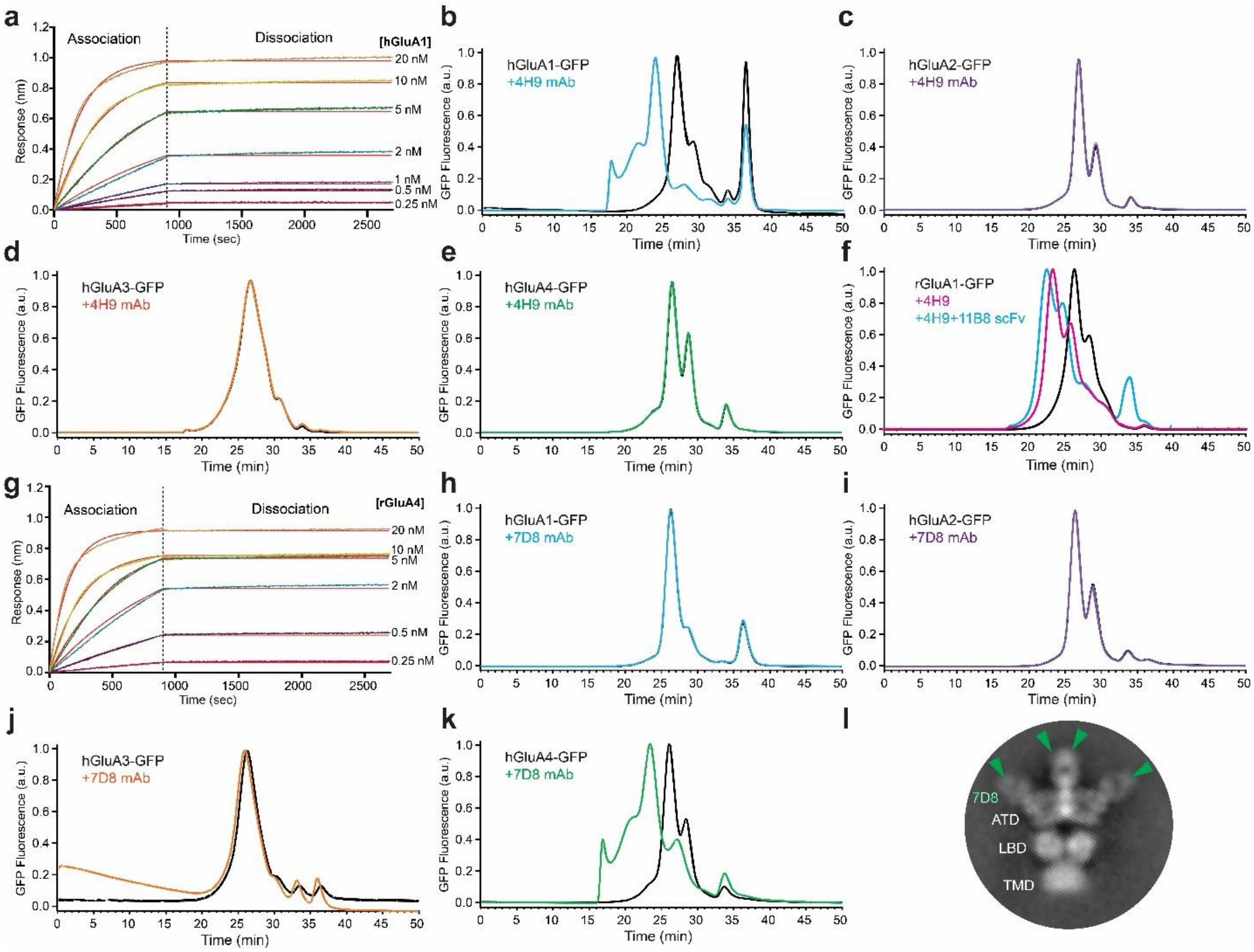

Supplementary Fig. 1. Characterization of monoclonal antibodies 4H9 and 7D8. (a) Octet measurements of the 4H9 monoclonal antibody binding to hGluA1, the Kd is ~5 nM. Concentrations of the hGluA1 ranging from 0.25 nM to 20 nM were applied. The fitting curves are colored red. (b-e) FSEC profiles of recombinant GFP-tagged hGluA1 (b), hGluA2 (c), hGluA3 (d), and hGluA4 (e) without 4H9 monoclonal antibody (black traces) and with 40 nM 4H9 monoclonal antibody (colored traces), detecting GFP fluorescence. Only hGluA1 receptors are shifted by the 4H9 monoclonal antibody. (f) The 4H9 monoclonal antibody can shift rGluA1 receptors and possesses a distinct epitope compared to the 11B8 monoclonal antibody. (g) Octet measurements of the 7D8 monoclonal antibody binding to rGluA4, the Kd is ~2 nM. Concentrations of the rGluA4 ranging from 0.25 nM to 20 nM were applied. The fitting curves are colored red. (h-i) FSEC profiles of recombinant GFP-tagged hGluA1 (h), hGluA2 (i), hGluA3 (j), and hGluA4 (k) without the 7D8 monoclonal antibody (black traces) and with 40 nM 7D8 monoclonal antibody (colored traces), detecting GFP fluorescence. Only hGluA4 receptors are shifted by the 7D8 monoclonal antibody. (l) The image shows a 2D class from single particle cryo-EM suggesting that the location of the epitope of 7D8 on rGluA4 is at the 'top' of the ATD. The sequence alignment of human and rat GluA1-4 in the ATD demonstrates an identity exceeding 99% (Supplementary Fig. 2). Therefore, the specificity of 4H9 and 7D8 in human AMPAR is likely to correspond to that in rats.

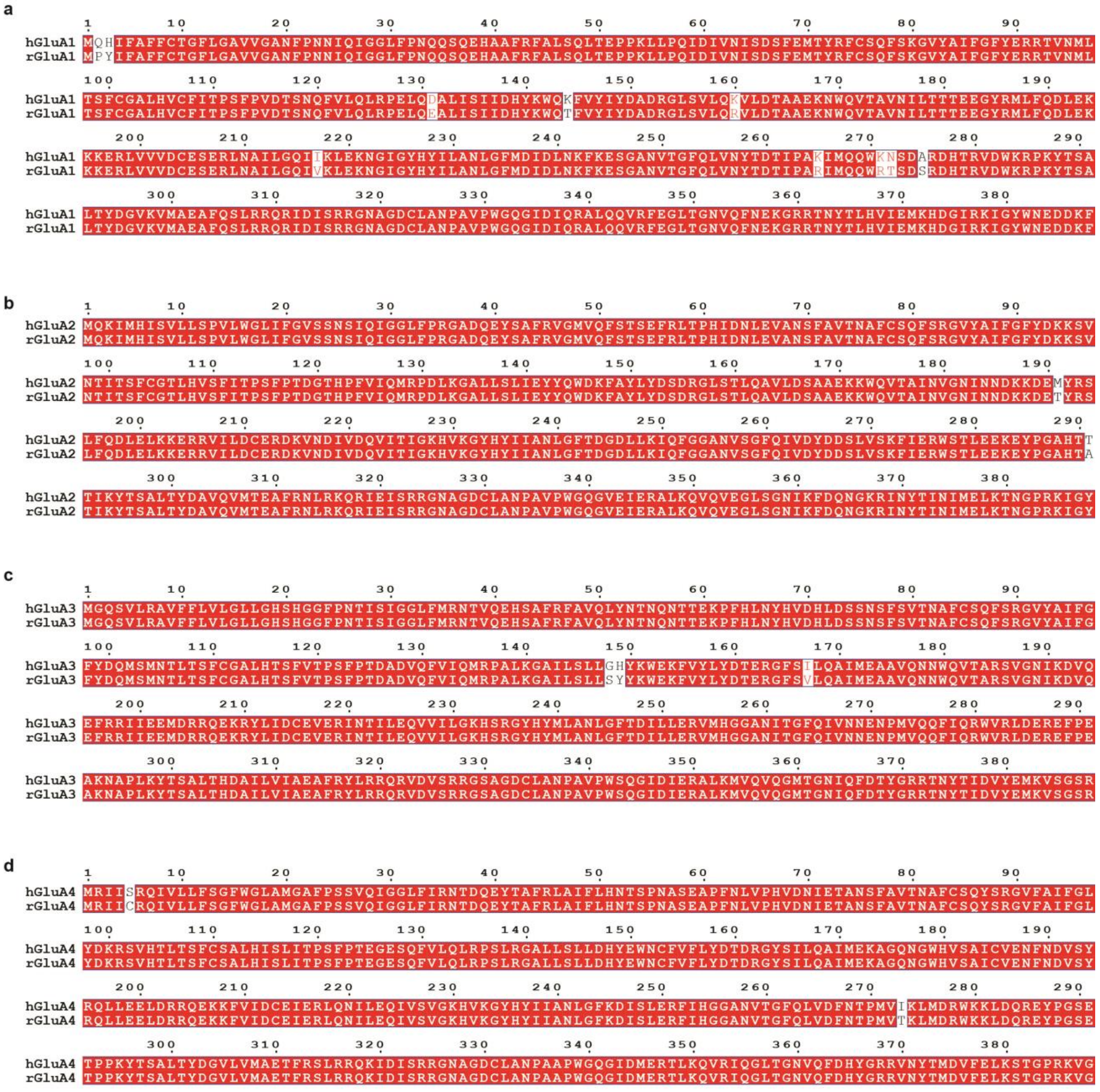

Supplementary Fig. 2. The sequence alignment of human GluA1-4 with rat GluA1-4.

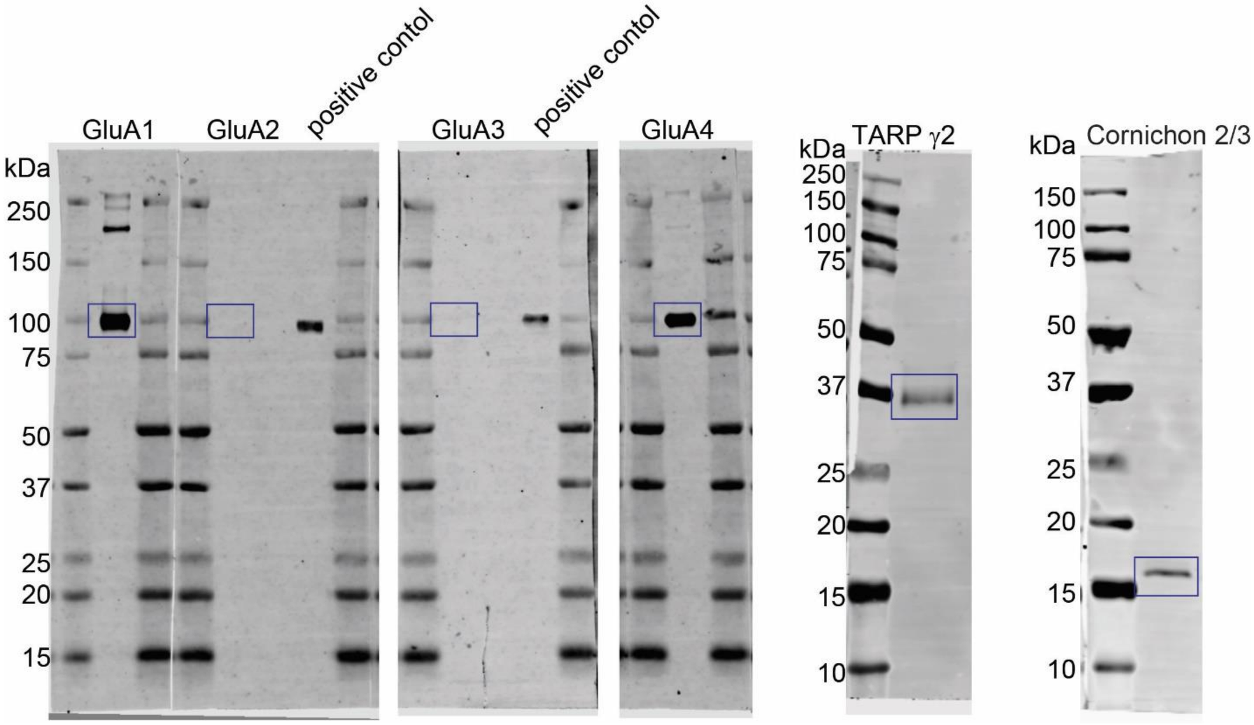

Supplementary Fig. 3. The raw western blot images. The GluA1, GluA2, GluA3, GluA4, TARP γ2, and cornichon 2/3 lanes were cropped to make Fig. 1b.

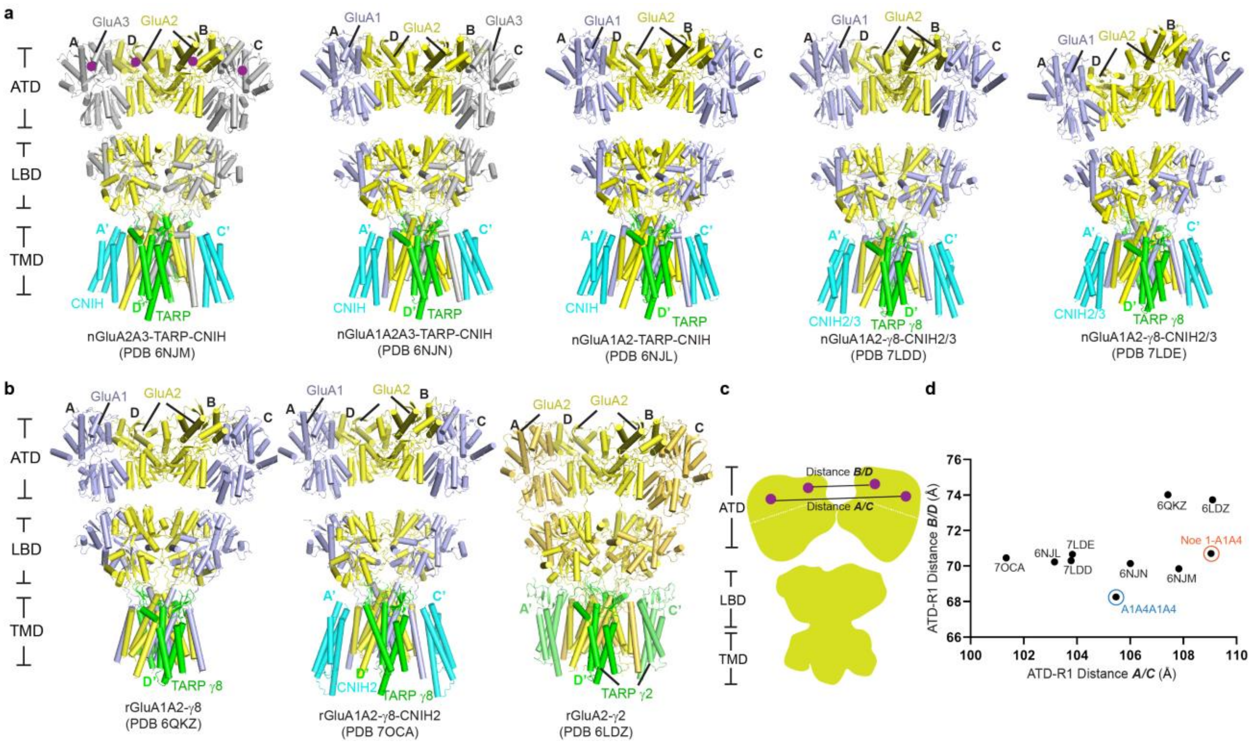

Supplementary Fig. 4. The structures of GluA2-containing AMPARs in complexes with auxiliary subunits. (a-b) The overall structures of GluA2-containing AMPARs from native tissue and recombinant sources. They show that GluA2-containing AMPARs harbor an ordered ATD layer compared with the CP-AMPARs in this study (Fig. 2), which have multiple conformations of ATD layer. (c-d) The analysis of GluA2-containing AMPARs in previous studies and GluA1A4 complexes with ordered ATD layer in this study. Purple points represent the center of mass (COM) of each subunit of ATD-R1 domain, respectively. The distance ***B/D*** represents the distance between the COM of ATD-R1 domain of subunits B and D. The distance ***A/C*** represents the distance between the COM of ATD-R1 domain of subunits A and C. (d) The plot of distance ***B/D*** and ***A/C***. In GluA2-containing AMPARs, GluA2 consistently occupies the B/D positions. In CP-AMPARs, GluA4 predominantly occupies the B/D positions, yet GluA1 also can occupy the B/D positions (Fig. 4). CI- and CP-AMPARs have similar auxiliary subunit occupancy where TARPs consistently predominate at the B'/D′ positions, and at the A'/C' positions there is greater auxiliary subunit heterogeneity.

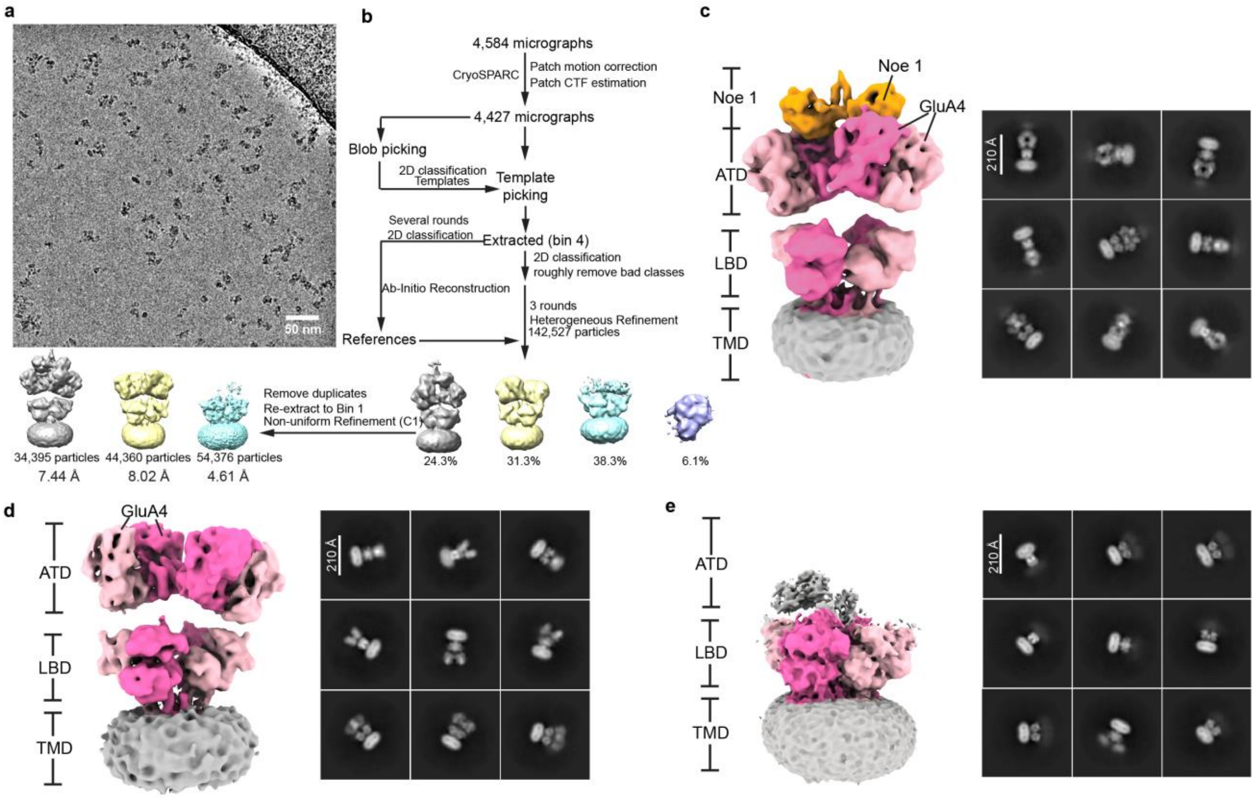

Supplementary Fig. 5. Cryo-EM analysis of recombinant GluA4 with Noe 1. (a) Representative motion-corrected micrograph. (b) Cryo-EM data processing workflow. (c) The 3D reconstruction and 2D class averages of Noe 1 with GluA4. (d) The 3D reconstruction and 2D class averages of GluA4 with an ordered ATD layer. (e) The 3D reconstruction and 2D class averages of GluA4 with a disordered ATD layer.

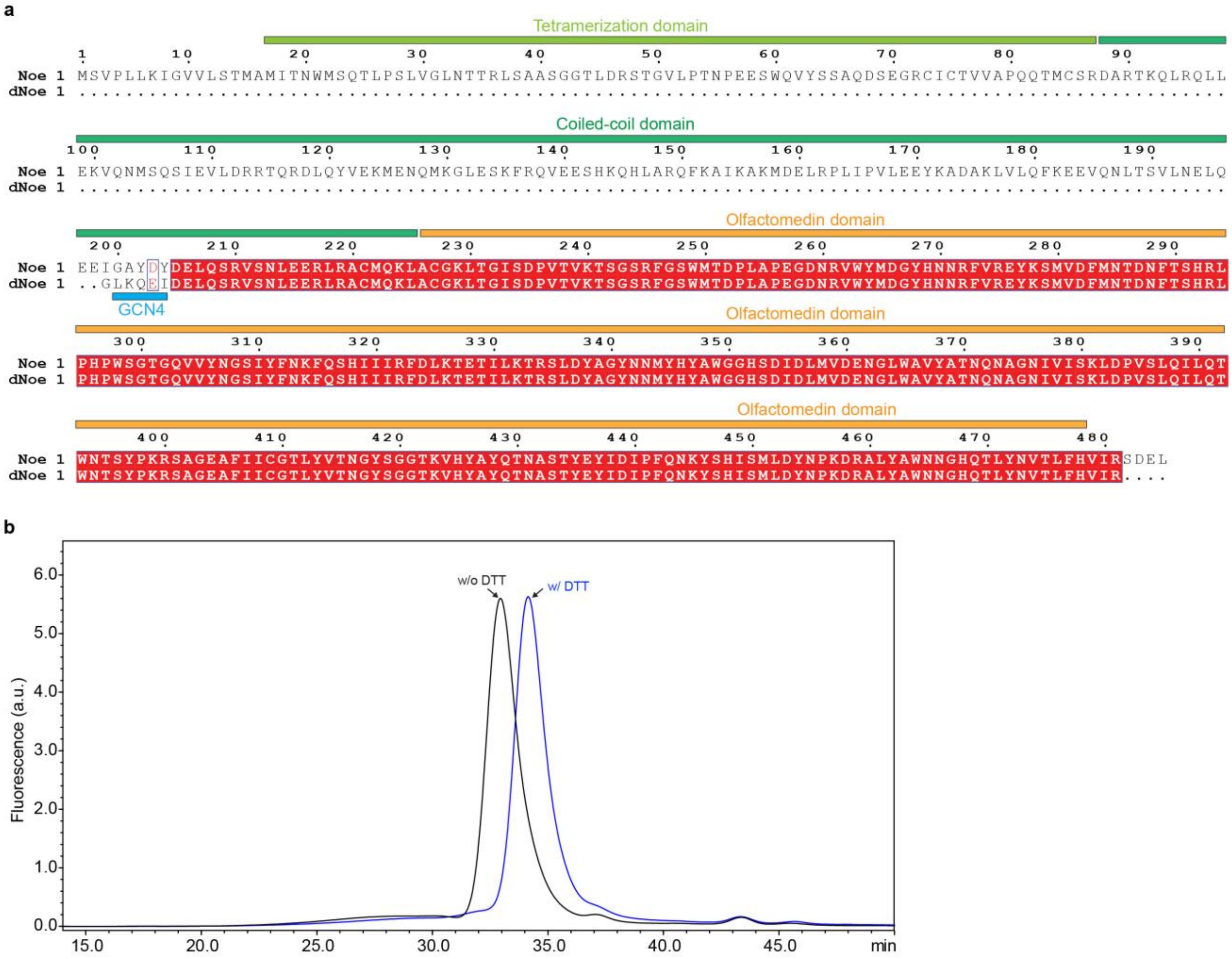

Supplementary Fig. 6. The sequence and FSEC profile for the engineered dimer of Noe 1 protein (dNoe 1).

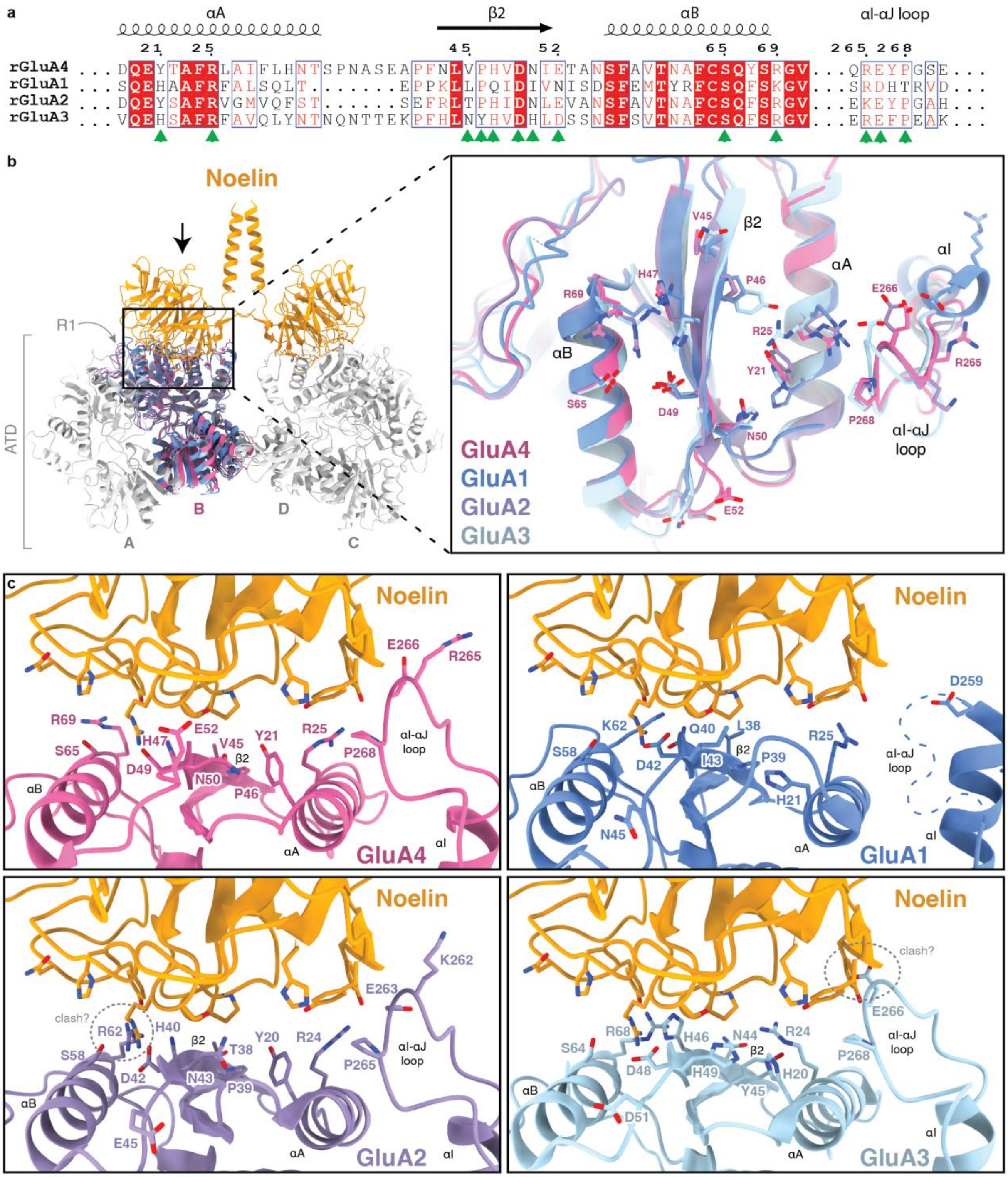

Supplementary Fig. 7. GluA1-4 alignments at Noe 1 interface. (a) Sequence alignment of residues of GluA1-4 receptors at the GluA4-Noe 1 interface, related to Extended Data Fig. 7. (b) Structure alignment of GluA1 (PDB 3SAJ), GluA2 (PDB 3HSY), GluA3 (PDB 3O21) ATD with GluA4 ATD at the Noelin 1 binding interface in the Noe 1-AMPAR complex (left) and a top-down view of the Noe1-GluA4 interface, highlighting key residues (right). (c) Individual views of hypothetical or modeled Noe1-GluAs interfaces shown in (b) with the equivalent, experimentally determined Noe1-GluA4 interface residues in GluA1-3 highlighted. The αI-αJ loop in GluA1

(PDB 3SAJ) and R265 in GluA3 (PDB 3O21) are not modeled and therefore not shown in alignments. Potential clashes evident between Noe 1 and GluA2 R62 or GluA3 E266 are noted.

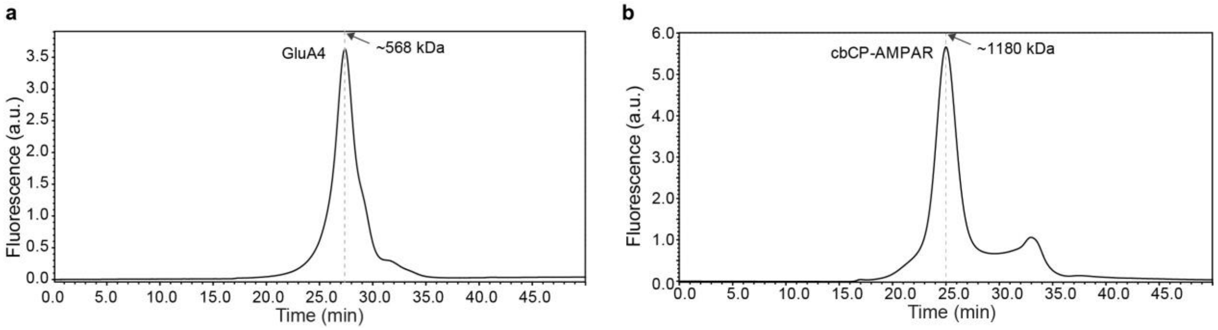

Supplementary Fig. 8. The FSEC profile for recombinant GluA4 and cerebellar CP-AMPAR, relative to Fig. 6.

Supplementary Table 1. The constructs in this study.

Construct	Protein
pFastBac-11B8 scFv-GFP-3C-Twin Strep	Anti-GluA1 11B8 scFv
pFastBac-15F1 Fab-mCherry-3C-Twin Strep	Anti-GluA2 15F1 Fab
pFastBac-L21-32R Fab-mCherry-3C-Twin Strep	Anti-GluA2 L21-32R Fab
pEGBacMam-Noe 1-Strep	Rat Noelin 1, Q62609, 1-485
pEGBacMam-Noe 1-mScarlet-His	Rat Noelin 1, Q62609, 1-485
pEGBacMam-dNoe 1-mScarlet-His	Rat Noelin 1, Q62609, 205-481
pEGBacMam-Twin Strep-3C-dNoe 1-mScarlet-His	Rat Noelin 1, Q62609, 205-481
pEGBacMam-IgGFc-dNoe 1-mScarlet-His	Rat Noelin 1, Q62609, 205-481
pEGBacMam-rGluA1-mScarlet-Flag	Rat GluA1, P19490, flip
pEGBacMam-rGluA4-GFP-3C-Twin Strep	Rat GluA4, P19493, flip
pEGBacMam-rGluA4-GFP-3C-Strep	Rat GluA4, P19493, flip
pEGBacMam-rGluA1-GFP-Strep	Rat GluA1, P19490, flip
pEGBacMam-rGluA2-GFP-Strep	Rat GluA2, P19491, flip, R
pEGBacMam-rGluA3-ΔC-GFP-Strep	Rat GluA3, P19492, flip, lacking C-terminal
pEGBacMam-rGluA4-ΔC-GFP-Strep	Rat GluA4, P19493, flip, lacking C-terminal
pEGBacMam-hGluA1-ΔC-GFP-Strep	Human GluA1, P42261, flip lacking C-terminal
pEGBacMam-hGluA2-ΔC-GFP-Strep	Human GluA2, P42262, flip, Q607R, lacking C-terminal
pEGBacMam-hGluA3-ΔC-GFP-Strep	Human GluA3, P42263, flip, R775G, lacking C-terminal
pEGBacMam-hGluA4-ΔC-GFP-Strep	Human GluA4, P42264, flip, R765G, lacking C-terminal
pEGBacMam-hGluA1-ΔC-Strep	Human GluA1, P42261, flip, lacking C-terminal
pEGBacMam-rGluA4-ΔC-Strep	Rat GluA4, P19493, flip, lacking C-terminal
