# Supplementary material for: Gating and noelin clustering of native Ca2+-permeable AMPA receptors
Source: Nature. 2025 Jun 23;645(8080):526–34. doi: 10.1038/s41586-025-09289-0 (PMC12422955; doi:10.1038/s41586-025-09289-0)
Supplement: Supplementary file 2 — Reporting Summary [file 41586_2025_9289_MOESM2_ESM.pdf]

nature portfolio

Corresponding author(s): Eric Gouaux

Last updated by author(s): Jun 9, 2025

Reporting Summary

Nature Portfolio wishes to improve the reproducibility of the work that we publish. This form provides structure for consistency and transparency in reporting. For further information on Nature Portfolio policies, see our Editorial Policies and the Editorial Policy Checklist.

Statistics

For all statistical analyses, confirm that the following items are present in the figure legend, table legend, main text, or Methods section.

n/a	Confirmed	
☐	☒	The exact sample size (*n*) for each experimental group/condition, given as a discrete number and unit of measurement
☐	☒	A statement on whether measurements were taken from distinct samples or whether the same sample was measured repeatedly
☐	☒	The statistical test(s) used AND whether they are one- or two-sided<br>*Only common tests should be described solely by name; describe more complex techniques in the Methods section.*
☒	☐	A description of all covariates tested
☐	☒	A description of any assumptions or corrections, such as tests of normality and adjustment for multiple comparisons
☐	☒	A full description of the statistical parameters including central tendency (e.g. means) or other basic estimates (e.g. regression coefficient) AND variation (e.g. standard deviation) or associated estimates of uncertainty (e.g. confidence intervals)
☐	☒	For null hypothesis testing, the test statistic (e.g. *F*, *t*, *r*) with confidence intervals, effect sizes, degrees of freedom and *P* value noted<br>*Give P values as exact values whenever suitable.*
☒	☐	For Bayesian analysis, information on the choice of priors and Markov chain Monte Carlo settings
☒	☐	For hierarchical and complex designs, identification of the appropriate level for tests and full reporting of outcomes
☒	☐	Estimates of effect sizes (e.g. Cohen's *d*, Pearson's *r*), indicating how they were calculated

Our web collection on statistics for biologists contains articles on many of the points above.

Software and code

Policy information about availability of computer code

Data collection	SerialEM 3.7, pClamp 10, Octet software 10.0 (Sartorius)
Data analysis	GraphPad Prism 10, CryoSPARC v4.4, COOT 0.9, Phenix 1.20, Chimera 1.16, Pymol 3.1, ChimeraX 1.6, Clamp fit 11.2, Octet software 10.0 (Sartorius)

For manuscripts utilizing custom algorithms or software that are central to the research but not yet described in published literature, software must be made available to editors and reviewers. We strongly encourage code deposition in a community repository (e.g. GitHub). See the Nature Portfolio guidelines for submitting code & software for further information.

Data

Policy information about availability of data

All manuscripts must include a data availability statement. This statement should provide the following information, where applicable:

- Accession codes, unique identifiers, or web links for publicly available datasets
- A description of any restrictions on data availability
- For clinical datasets or third party data, please ensure that the statement adheres to our policy

The cryo-EM maps and coordinates for the Noelin-GluA1/A4-ATD and Noelin-GluA1/A4 LBD-TMD have been deposited in the Electron Microscopy Data Bank (EMDB) under accession numbers EMD-49723 and EMD-49724 and in the Protein Data Bank (PDB) under accession codes 9NR6 and 9NR7, respectively. The cryo-EM maps and coordinates for the GluA1/A4-ATD, GluA1/A4 LBD-TMD with 4 auxiliary proteins, and GluA1/A4 LBD-TMD with 2 TARPs have been deposited in EMDB

under accession numbers EMD-49725, EMD-49727, and EMD-49726 and in PDB under accession codes 9NR8, 9NRA, and 9NR9, respectively. The cryo-EM maps for the LBD-TMDmix-4TARPs, LBD-TMDmix-2TARPs, LBD-TMDmix-2TA.RPs-2CNIHs, Noelin-GluA1/A4, GluA1/A4 with ordered ATD and disordered ATD have been deposited in EMDB under accession numbers EMD-49711, EMD-49712, EMD-49713, EMD-49714, EMD-49715, and EMD-49716, respectively. The cryo-EM map for the LBD-TMD with 4 auxiliary subunits in the active state has been deposited in EMDB under accession numbers EMD-49717. The cryo-EM maps for the GluA1/A4 LBD-TMD and Noelin-GluA1/A4 LBD-TMD in the desensitized state have been deposited in the EMDB under accession numbers EMD-49718 and EMD-49719, respectively. The cryo-EM maps for the recombinant Noelin-GluA4, GluA4 with ordered ATD and disordered ATD have been deposited in EMDB under accession numbers EMD-49722, EMD-49720, and EMD-49721, respectively. The reference models of 7LEP and 7LDD used for model building were obtained from the PDB.

Research involving human participants, their data, or biological material

Policy information about studies with human participants or human data. See also policy information about sex, gender (identity/presentation), and sexual orientation and race, ethnicity and racism.

Reporting on sex and gender	N/A
Reporting on race, ethnicity, or other socially relevant groupings	N/A
Population characteristics	N/A
Recruitment	N/A
Ethics oversight	N/A

Note that full information on the approval of the study protocol must also be provided in the manuscript.

Field-specific reporting

Please select the one below that is the best fit for your research. If you are not sure, read the appropriate sections before making your selection.

☒ Life sciences ☐ Behavioural & social sciences ☐ Ecological, evolutionary & environmental sciences

For a reference copy of the document with all sections, see nature.com/documents/nr-reporting-summary-flat.pdf

Life sciences study design

All studies must disclose on these points even when the disclosure is negative.

Sample size	For cryo-EM experiments, sample sizes were determined by the availability of microscope time and the number of high-quality particles obtained. The final particle numbers were sufficient to achieve the reported resolution, as validated by Fourier shell correlation (FSC) analysis. For single-molecule pull-down (SiMPull) experiments, at least 15 images were collected, which provided reproducible results across replicates. Electrophysiology recordings were repeated at least 14 times using different cells. The sample sizes for both SiMPull and electrophysiology experiments were determined based on the observed consistency and variability of the data, informed by prior literature and our previous experience. Overall, the sample sizes were sufficient to support the robustness and reproducibility of the reported findings.
Data exclusions	No data were excluded from the analyses.
Replication	Cryo-EM–related experiments, including protein purification and FSEC, were independently reproduced at least three times with consistent results. SDS-PAGE, Western blot, and mass spectrometry analyses were independently repeated twice, yielding reproducible outcomes. Electrophysiology recordings were performed on at least 14 different cells, and all replicates produced consistent responses. Radioligand binding assays were carried out in three parallel trials, all of which yielded comparable binding profiles. Octet experiments were independently repeated three times, with consistent kinetic parameters observed across replicates. SiMPull experiments were performed by collecting at least 17 images, and all replicates yielded reproducible patterns.
Randomization	For cryo-EM data processing, particle datasets were randomly divided into two halves following the standard gold-standard refinement procedure implemented in cryoSPARC. Group allocation was not applicable to SiMPull experiments, cryo-EM–related biochemical experiments, electrophysiology, Octet experiments, and radioligand binding assays, as these experiments did not involve predefined experimental groups or treatment conditions. For SiMPull experiments, images were acquired from randomly selected regions of the sample chamber to avoid selection bias. Cryo-EM–related biochemical experiments, including protein purification and FSEC, were repeated using independently prepared samples from different batches of rat cerebellum. For electrophysiology recordings, GFP-positive cells were randomly selected for patching, without prior knowledge of their electrophysiological properties. Octet and radioligand binding assays were performed on uniform sample preparations and thus did not require further group allocation.
Blinding	The investigators were not blinded. Blinding was not applicable to cryo-EM data collection and analysis, cryo-EM related biochemical experiments, and mass spectrometry analyses, because this type of study does not use group allocation. It is not technically or practically feasible to do so for electrophysiology recording, Octet experiments, FSEC experiments, radioligand binding assays or Single molecule pull down experiments.

Reporting for specific materials, systems and methods

We require information from authors about some types of materials, experimental systems and methods used in many studies. Here, indicate whether each material, system or method listed is relevant to your study. If you are not sure if a list item applies to your research, read the appropriate section before selecting a response.

Materials & experimental systems

n/a	Involved in the study
☐	☒ Antibodies
☐	☒ Eukaryotic cell lines
☒	☐ Palaeontology and archaeology
☐	☒ Animals and other organisms
☒	☐ Clinical data
☒	☐ Dual use research of concern
☒	☐ Plants

Methods

n/a	Involved in the study
☒	☐ ChIP-seq
☒	☐ Flow cytometry
☒	☐ MRI-based neuroimaging

Antibodies

Antibodies used	11B8 scFv anti-GluA1 (produced by our lab); 15F1 Fab anti-GluA2 (produced by our lab); 5B2 Fab anti-GluA3 (produced by our lab);7D8 anti-GluA4 (produced by our lab), 4H9 anti-GluA1 (produced by our lab), L21-32R Fab anti-GluA2 (DNA sequence from addgene, https://www.addgene.org/177480/), anti-GFP nanobody (plasmid of the GFP nanobody was a gift from Brett Collins, and was expressed, purified in our lab). Commercial antibodies: anti-GluA1 (Millipore, N453, 04-823,), anti-GluA2 (Thermo Fisher, N/A, PAS-19496), anti-GluA3 (Invitrogen,3B3,32-0400), anti-GluA4 (Millipore, N/A, ab1508). We have stated in the method that the antibodies used for structural determination were not diluted, antibodies used for single molecule pull down experiments were diluted to at a concentration of 10 to 30 ug/ml and antibodies used for Western Blot were diluted in1: 1000. IRDye 800 CW anti-mouse/rabbit secondary antibodies were used for western blot visualization. Blots were developed by adding secondary antibodies at a ratio of 1:10,000.
Validation	Validation of 11B8 scFv anti-GluA1, 5F1 Fab anti-GluA2 and 5B2 Fab anti-GluA3 used for cryo-EM structure determination and single molecule pull down experiments can be found in the previous published literature (Zhao, Y. et al. Architecture and subunit arrangement of native AMPA receptors elucidated by cryo-EM. Science 364, 355-362 (2019)). The validation of anti-GluA1 4H9 and anti-GluA4 7D8 could be found in the Supplementary Fig. 1. The validation of L21-32R anti-GluA2 could be found in the website:https://www.addgene.org/177480/. The refernece for the GFP nanobody is : Kubala, M. et al. Structural and thermodynamic analysis of the GFP:GFP-nanobody complex. Protein Sci. 2010 Dec;19(12):2389-401. The validation of commercial antibodies anti-GluA1, anti-GluA2, anti-GluA3, and anti-GluA4 for western blot could be found in the websites: https://www.emdmillipore.com/US/en/product/Anti-phospho-GluR1-Ser831-Antibody-clone-N453-rabbit-monoclonal, MM_NF-04-823?ReferrerURL=https%3A%2F%2Fwww.google.com%2F; https://www.thermofisher.com/antibody/product/GluR2-Antibody-Polyclonal/PAS-19496; https://www.thermofisher.com/antibody/product/GluR3-Antibody-clone-3B3-Monoclonal/32-0400; https://www.emdmillipore.com/US/en/product/Anti-Glutamate-Receptor-4-Antibody, MM_NF-AB1508, respectively.

Eukaryotic cell lines

Policy information about cell lines and Sex and Gender in Research

Cell line source(s)	Sf9 cells for expression of Baculovirus are from Thermofisher (12659017, lot 421973). HEK2935S GnTI- cells for protein expression and electrophysiology studies were purchased from ATCC (CRL-3022). The tsA201 cells for the expression of AMPARs are purchase form ATCC (CRL-11268).
Authentication	The cells were routinely maintained in our lab. They were not authenticated experimentally for these studies.
Mycoplasma contamination	Sf9 cells,tsA 201 cells and HEK293S GnTI- cells were tested negative.
Commonly misidentified lines (See ICLAC register)	No commonly misidentified lines were applied.

Animals and other research organisms

Policy information about studies involving animals; ARRIVE guidelines recommended for reporting animal research, and Sex and Gender in Research

Laboratory animals	The study did not involve laboratory animals, rat carcasses donated from other laboratories of OHSU.
Wild animals	The study did not involve wild animals.
Reporting on sex	The cerebellum tissue derived from male and female Rattus norvegicus rats

Field-collected samples | No filed-collected samples were used in this study.

Ethics oversight | All rats were euthanized under the OHSU Institutional Animal Care and Use Committee (IACUC) protocols, consistent with the recommendations of the Panel on Euthanasia of the American Veterinary Medical Association (AVMA).

Note that full information on the approval of the study protocol must also be provided in the manuscript.

Plants

Seed stocks | *Report on the source of all seed stocks or other plant material used. If applicable, state the seed stock centre and catalogue number. If plant specimens were collected from the field, describe the collection location, date and sampling procedures.*

Novel plant genotypes | *Describe the methods by which all novel plant genotypes were produced. This includes those generated by transgenic approaches, gene editing, chemical/radiation-based mutagenesis and hybridization. For transgenic lines, describe the transformation method, the number of independent lines analyzed and the generation upon which experiments were performed. For gene-edited lines, describe the editor used, the endogenous sequence targeted for editing, the targeting guide RNA sequence (if applicable) and how the editor was applied.*

Authentication | *Describe any authentication procedures for each seed stock used or novel genotype generated. Describe any experiments used to assess the effect of a mutation and, where applicable, how potential secondary effects (e.g. second site T-DNA insertions, mosiacism, off-target gene editing) were examined.*
